# Transiently Nav1.8-expressing neurons are capable of sensing noxious stimuli in the brain

**DOI:** 10.3389/fncel.2022.933874

**Published:** 2022-08-29

**Authors:** Helia Tenza-Ferrer, Mélcar Collodetti, Eduardo de Souza Nicolau, Alexander Birbrair, Luiz Alexandre Viana Magno, Marco Aurélio Romano-Silva

**Affiliations:** ^1^Centro de Tecnologia em Medicina Molecular, Universidade Federal de Minas Gerais (UFMG), Belo Horizonte, Brazil; ^2^Departamento de Patologia, Instituto de Ciências Biológicas, Universidade Federal de Minas Gerais (UFMG), Belo Horizonte, Brazil; ^3^Department of Dermatology, University of Wisconsin-Madison, Madison, WI, United States; ^4^Department of Radiology, Columbia University Medical Center, New York, NY, United States; ^5^Curso de Medicina, Universidade José do Rosário Vellano (UNIFENAS), Belo Horizonte, Brazil; ^6^Pós-graduação da Faculdade Ciências Médicas de Minas Gerais, Belo Horizonte, Brazil; ^7^Departamento de Saúde Mental, Faculdade de Medicina, Universidade Federal de Minas Gerais (UFMG), Belo Horizonte, Brazil

**Keywords:** Nav1.8, limbic circuitry, central nervous system, inflammatory pain, neurons, cFOS, neurocircuit, amygdala

## Abstract

While current research highlights the role of Nav1. 8 sensory neurons from the peripheral nervous system, the anatomical and physiological characterization of encephalic Nav1.8 neurons remains unknown. Here, we use a Cre/fluorescent reporter mouse driven by the Nav1.8 gene promoter to reveal unexpected subpopulations of transiently-expressing Nav1.8 neurons within the limbic circuitry, a key mediator of the emotional component of pain. We observed that Nav1.8 neurons from the bed nuclei of the stria terminalis (BST), amygdala, and the periaqueductal gray (vPAG) are sensitive to noxious stimuli from an experimental model of chronic inflammatory pain. These findings identify a novel role for central Nav1.8 neurons in sensing nociception, which could be researched as a new approach to treating pain disorders.

## Introduction

Nav1.8 is a voltage-gated sodium channel (VGSC) that is important for neuronal excitability in nociception. Alterations in Nav1.8 sodium current in sensory neurons affect normal pain-signaling and underlie the pathophysiology of pain states, such as neuropathic and chronic inflammatory pain. For example, an early report showed that intrathecal administration of Nav1.8 mRNA antisense oligonucleotides reversed the neuropathic pain (Lai et al., 2002). Moreover, thermal hyperalgesia was abolished in Nav1.8-knockout mice, supporting the role of Nav1.8 in the inflammatory pain (Kerr et al., 2001).

While most research highlights the role of Nav1.8 sensory neurons from the dorsal root ganglia (DRG) (Hudmon et al., 2008; Shields et al., 2012b), the identity and physiology of encephalic Nav1.8 neurons remain unclear. This fact may be due to the belief that Nav1.8 channels express selectively in the peripheral nervous system (PNS) (Shields et al., 2012a). However, recent studies using transgenic mice suggest otherwise. For example, Nav1.8-Cre-tdT mice contain positive neurons for the td-Tomato fluorescent protein (tdT) in the brain (Stirling et al., 2005), implying that CNS neurons exhibit Cre recombinase activity driven by the Nav1.8 gene promoter. Therefore, possibly, a whole-brain mapping of Nav1.8-Cre-tdT mice may uncover unanticipated functions of encephalic Nav1.8 neurons.

The central endeavor in neuroscience is not only to map the distribution but also the function of specific neuronal subpopulations. Therefore, new findings regarding the precise location of CNS Nav1.8 neurons and their relationship to neurocircuits may support experimental evidence for the proposed roles of Nav1.8 in pain. Thus, if these CNS Nav1.8 neurons also affect the processing of nociception in brain networks, their manipulation could be a new approach to counteract pain disorders.

Here, we have used Nav1.8-Cre-tdT mice to characterize the anatomical distribution, morphology, neurotransmitter phenotype, and the sensing of noxious stimuli of Nav1.8 neurons from the brain.

## Materials and methods

### Animals

Wild-type (WT) mice (C57Bl/6) were obtained from the UFMG Breeding Center (Belo Horizonte, Brazil). Nav1.8-Cre-tdT mice (Gautron et al., 2011) were maintained on a C57Bl/6 background at our facility. This mouse line was crossed to WT mice to generate Nav1.8-Cre mice. Gad2-Cre mice were obtained from Jackson Laboratories (Bar Harbor, ME). Experiments were performed with adult male or female mice aged between 8 and 12 weeks old. All the animals were housed in plastic cages in a humidity-controlled facility maintained on a 12-h light/dark cycle (lights on at 6:00 a.m.). All animals were kept with food and water available *ad libitum* throughout the experiments and were randomly allocated to the different experimental groups. All animal protocols were performed following National Institutes of Health (NIH) guidelines and approved by the Federal University of Minas Gerais (protocol 280/2019).

### Surgical procedures

Stereotaxic injections were performed as previously described (Magno et al., 2019). Briefly, Gad2-Cre or Nav1.8-Cre mice were anesthetized with an intraperitoneal injection of ketamine-xylazine (80 and 8 mg/kg, respectively) and placed into a stereotaxic frame (David Kopf Instruments). A mix of oxygen (1.0 L/min) and isoflurane at 1% was used to maintain a deeply anesthetized state for the surgery. Body temperature was controlled by a feedback heating pad throughout the procedure (David Kopf Instruments). The craniotomy was performed using a dental drill with a 0.75-mm burr. A pulled glass micropipette (Nanoliter 2010, World Precision Instruments) was used to inject the viruses into the orbital cortex (ORB), dorsal striatum (STRd), or central amygdala (CeA): +2.4 mm (AP), +0.35 mm (ML), −1.8 mm (DV) [ORB]; −1.55 mm (AP), +3.25 mm (ML), −3.5 mm (DV) [STdr]; and −1.55 mm (AP), +3.0 mm (ML) −4.9 mm (DV) [CeA]. We injected a total of 1,000 nL of viral suspension at an injection rate of 100 nL/min. These experiments were carried out using AAV5-Ef1a-DIO-eYFP (6.5 ×10^12^) (viral stock #27056-AAV5, Addgene). After injection, the micropipette was left in place for an additional 5 min and then slowly withdrawn. The skin was sealed with tissue adhesive (Vetbond, 3M) and ketoprofen (5 mg/kg) was injected subcutaneously one time daily for 3 days. Mice were allowed to recover for 4 weeks after virus injection.

### Brain dissection and RT-PCR

Mice were anesthetized with an intraperitoneal injection of ketamine-xylazine (100 and 10 mg/kg, respectively) and decapitated following cervical dislocation. Brains were quickly removed and sliced into 2–3-mm-thick coronal sections with disposable razor blades using an ice-cold mouse brain matrix cleaned with RNase Zap solution (Life Technologies). The cerebellum (Cer), STRd [including the lateral septum (LS)], and amygdala (Amyg) were identified using a mouse brain atlas (Allen, 2004) and dissected using a tissue puncher. We also dissected four dorsal root ganglia (DRG) from each mouse. All the samples were rapidly transferred into tubes filled with 500 μl of TRIzol^®^ reagent (Life Technologies) and frozen in dry ice before the cryopreservation at −80°C until the time of use. Total RNAs were extracted using the TRIzol^®^ method. Purified samples were qualified and quantified using a NanoDrop 2000 spectrophotometer (Life Technologies). RNA samples containing 500 ng of total RNAs were reverse-transcribed into complementary DNA (cDNA) as described elsewhere (Magno et al., 2020). PCRs were carried out using three primer sets designed using the Primer-BLAST software (Ye et al., 2012). We used the *Mus musculus Scna10* gene (Gene ID 20264) as a reference. A schematic and the sequences of the primer sets are given in **Figure 4** and Supplementary Table S1. The *Scn10a* transcripts are shown in Supplementary Table S2. The primer set exon 1-2 recognizes either the inclusion of exon 1 or the exon 1-2 junction, and thus, it can detect all the described Nav1.8 isoforms 1, 2, X1, X2, X3, and X4. Primer set exon 16 recognizes the exon 16 (also present in all isoforms), whereas the primer set exon 22–26 recognizes a region in exon 26 unique to the isoforms 1, 2, and X4. PCR was performed using 20% of the total synthesized cDNA with the GoTaq^®^ Master Mix (Promega).

PCRs were carried out in a T100™ thermocycler (Bio-Rad) with the following cycling conditions: 2 min at 95°C, followed by 40 cycles of 30 s at 94°C, 30 s at 60°C, and 30 s at 72°C. The reaction included a final extension step of 5 min at 72°C. The amplification products for each primer set were run on 1.5% agarose gels, stained with ethidium bromide, and photo-documented in UV light using the ImageQuant 400 (GE Healthcare Life Sciences) imaging system. In all cases, the DNA bands displayed the expected nucleotide length when compared to the DNA ladder.

### The experimental model of chronic pain

The experimental model of chronic pain was performed as described before (Tenza-Ferrer et al., 2019). Briefly, Freund's Complete Adjuvant (CFA; Sigma-Aldrich) consisted of 1 mg/ml heat-inactivated *Mycobacterium tuberculosis* in 85% paraffin oil and 15% mannan monooleate. Mice received a single subcutaneous intraplantar injection of 10 μl of CFA or phosphate-buffered saline (PBS) into both the right and left hind paws using a 27-gauge needle. Chronic pain was produced over 2 days to observe neuronal activity in the brain.

### Histology

Histology was performed as previously described (Magno et al., 2019). Mice were anesthetized with ketamine/xylazine (100 per 10 mg/kg body weight) and transcardially perfused with ice-cold PBS (pH 7.4), followed by freshly prepared 4% paraformaldehyde (PFA) in PBS. Brains were postfixed for 12 h in 4% PFA at 4°C. Coronal slices (50 or 100 μm thick) were prepared using a vibrating blade microtome (Leica Microsystems). The free-floating slices were washed with PBS and permeabilized with 0.1% Triton X-100 in PBS and blocked with 4% BSA for 1.5 h and incubated overnight at 4°C in the same solution containing the following primary antibodies at the indicated dilutions: anti-NeuN (Millipore Cat# ABN91, RRID: AB_11205760, 1:1,000), anti-tyrosine hydroxylase (Abcam Cat# ab76442, RRID: AB_1524535, 1:1,000), anti-c-Fos (Cell Signaling Cat# 2250, RRID: AB_2247211, 1:500), anti-VGluT1 (Millipore Cat# MAB5502, RRID: AB_262185, 1:500), anti-VGluT2 (Millipore Cat# AB2251-I, RRID: AB_2665454, 1:500), anti-Gad1/2 (Millipore Cat# ABN904, RRID: AB_2893025, 1:1,000), anti-ChAT (Millipore Cat# AB144P, RRID: AB_2079751, 1:1,000), anti-TPH2 (Millipore Cat# AB1541, RRID: AB_90754, 1:1,000), anti-GFP (Thermo Fisher Scientific Cat# A-11120, RRID: AB_221568, 1:2,000), anti-GFAP (Sigma-Aldrich, Cat# G3893, RRID: AB_477010, 1:400), anti-Iba1 (Wako, Cat# 019-19741, RRID: AB_839504, 1:400), and anti-mCherry (Abcam, Cat# AB167453, RRID: AB_2571870, 1:1,000). After primary antibody incubation, slices were rinsed three times in PBS for 10 min each time and incubated for 2 h with the following Alexa Fluor (AF) fluorophore-conjugated secondary antibodies: AF-488 donkey anti-rabbit (Thermo Fisher, Cat# A21206, RRID: AB_2534073, 1:1,000), AF-594 goat anti-rabbit (Thermo Fisher, Cat# A11012, RRID: AB_2534079, 1:1,000), AF-647 goat anti-rabbit (Thermo Fisher, Cat# A21244, RRID: AB_2535812, 1:1,000), AF-647 goat anti-chicken (Thermo Fisher, Cat# A21449, RRID: AB_2535866, 1:1,000), AF-488 goat anti-guinea pig (Thermo Fisher, Cat# A11073, RRID: AB_2534117, 1:1,000), AF-488 goat anti-mouse (Thermo Fisher, Cat# A11029, RRID: AB_2534088, 1:1,000), AF-647 donkey anti-goat (Thermo Fisher, Cat# A21447, RRID: AB_2535864, 1:1,000), and AF-488 donkey anti-sheep (Thermo Fisher, Cat# A11015, RRID: AB_2534082, 1:1,000). Following incubation, the slices were washed three times with PBS and mounted in Dako fluorescent mounting medium (Agilent Technologies).

We investigated every coronal section (100 μm thick) in the full anterior–posterior (AP) span of the brain by searching slices containing fluorescent tdT+ (Nav1.8+) cell bodies mapped to the Allen Brain Atlas (Allen, 2004). We concluded that these neurons were found only within slices containing the lateral septal nucleus (LS), bed nuclei of the stria terminalis (BST), dorsal striatum (STRd), amygdala (Amyg), hypothalamus (HY), and the ventral periaqueductal gray (vPAG). We then co-labeled tdT+ slices with anti-mCherry (for tdT) and anti-NeuN (for neuronal nuclei) antibodies and used a Leica SP5 confocal microscope equipped with a motorized XY-stage (Leica Microsystems) to acquire confocal z-stacks covering a depth of 30 μm. All unsaturated images were captured under identical conditions (20X or 63X objective, resolution of 1,024 × 1,024 pixels, and 200 Hz speed). To study the neuronal spatial distribution in larger brain areas, 20X images were acquired in individual z-projections and stitched together to produce an entire region using the LAS (Leica Microsystems) or Fiji/ImageJ (Schindelin et al., 2012) software.

To quantify the number of Nav1.8+ or NeuN+ cell bodies in the brain, we performed double immunofluorescence (as described above) and manually counted the cells in the selected brain areas. The regions of interest (ROIs) were built in Fiji (Schindelin et al., 2012) guided by the Allen Mouse Brain Atlas (Allen, 2004). Nav1.8 density is the ratio between tdT and NeuN counts. The number or density of cFos+ neurons following the chronic pain experiment was quantified using the same procedure. All counts were conducted by at least two experimenters blinded to the treatment group. To analyze the potential co-localization of Nav1.8 neurons with the neurotransmitter markers (Gad1/2, vGlut1/2, ChAT, TPH2, or TH), we selected the representative coronal slices with the highest Nav1.8 density from two mice. These analyses were performed as double fluorescent protocols using each time a single neurotransmitter marker tagged with an AF-488 secondary antibody and tdT. For each brain region, acquisitions were performed in at least 4 different fields containing both fluorescences with the help of a motorized XY-stage guided by the Mark and Find multiple position tool (Leica Microsystems). Every z-stack captured with a 63X oil immersion objective was analyzed slice by slice with the orthogonal projection tool in Fiji software to investigate the potential co-localization on the same plane. The perisomatic morphology was analyzed in z-stacks converted to binary images and edited manually to clear the background. The number of the primary and secondary dendrites was manually counted. The soma size was measured in binary images containing only the cell soma using the analyze particles tool from Fiji.

### Experimental design and statistical analyses

For all datasets, normality was tested using the D'Agostino-Pearson omnibus test (alpha < 0.05) and homogeneity of variance with Brown–Forsythe's test (alpha < 0.05) to determine whether parametric or nonparametric analyses were required. For two-sample comparisons of a single variable, we used the unpaired Student's *t*-test. The experimenter was blinded to the experimental groups while running the statistical analyses. No statistical methods were used to predetermine sample sizes, but our sample sizes are like those reported in previous publications (Kravitz et al., 2010; Kim et al., 2017). For the exact number of animals used in each experiment and details of statistical analyses, refer to the results. All tests were two-tailed and had an alpha level of 0.05. All statistical analyses were performed using GraphPad Prism version 6 (GraphPad Software).

## Results

### Spatial distribution of Nav1.8 neurons in the brain

**Figure 1:**
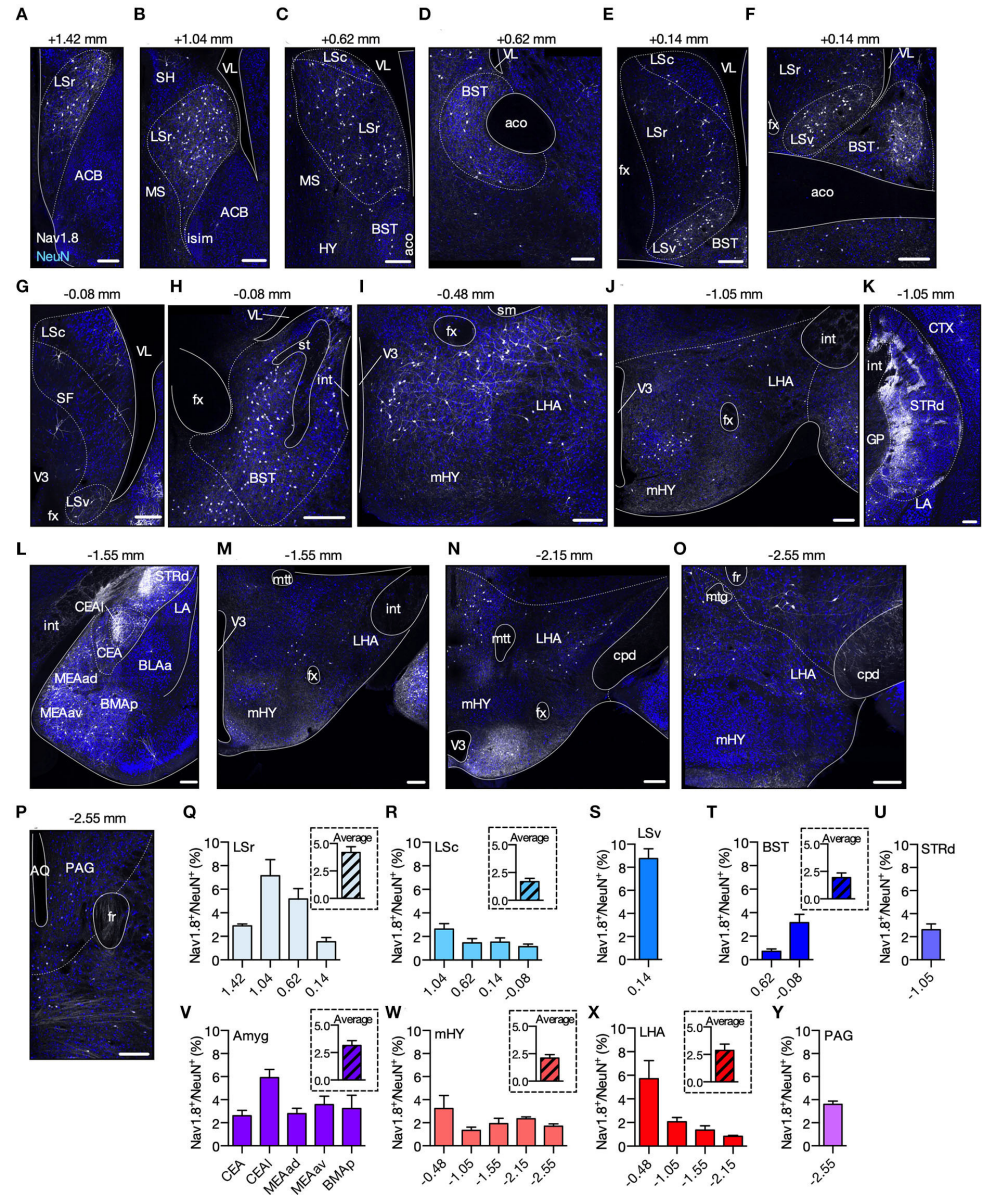
Spatial distribution of Nav1.8+ neurons in the brain. **(A–P)** Representative coronal sections containing tdT fluorescence from Nav1.8+ neurons in Nav1.8-Cre-tdT mice. The number on the top of each image shows the position relative to the bregma. Gray, tdT fluorescence; blue, NeuN from immunoreactivity. **(Q–Y)** Density of Nav1.8+ cell bodies (relative to NeuN) (*n* = 4; 2 males and 2 females). In the inset, average density for the brain areas where analyses were performed in multiple anterior-posterior locations. ACB, nucleus accumbens; aco, anterior commissure, olfactory limb; AQ, aqueduct; BLAa, basolateral amygdalar nucleus, anterior part; BMAp, basomedial amygdalar nucleus, posterior part; BST, bed nuclei of the stria terminalis; Cc, corpus callosum; CEA, central amygdalar nucleus; CEAl, central amygdalar nucleus, lateral part; cpd, cerebral peduncle; CTX, cerebral cortex; fr, fasciculus retroflexus; fx, columns of the fornix; GP, globus pallidus; int, internal capsule; islm, major island of Calleja; LA, lateral amygdalar nucleus; LHA, lateral hypothalamic area; LSc, LSr and LSv, caudal, rostral and ventral part of the lateral septal nucleus; MEAad, MEAav, medial amygdalar nucleus, anterodorsal (ad) or anteroventral (av) part; mHY, medial hypothalamus; MS, medial septal nucleus; mtt, mammillothalamic tract; PAG, periaqueductal gray; SH, septohippocampal nucleus; sm, stria medullaris; st, stria terminallis; STRd, striatum dorsal region; TH, thalamus; V3, third ventricle; VL, lateral ventricle. Summary data are represented as mean ± SEM. Scale bar = 200 μm.

Nav1.8-Cre-tdT mice express the td-Tomato fluorescent protein (tdT) following Cre recombinase expression driven by the Nav1.8 gene promoter (Stirling et al., 2005). In the first experiment, our goal differed from that of previous studies, where tdT-positive neurons (Nav1.8+) in Nav1.8-Cre-tdT mice were examined in tissues other than the brain nor mapped to predict the precise neuronal locations (Gautron et al., 2011). Our strategy was to search for Nav1.8+ cell bodies in the full anterior–posterior (AP) span of the brain by imaging confocal z-stacks covering a depth of 30 μm of every second tdT+ coronal sections (50 μm thick) colabeled with NeuN. Nav1.8+ and NeuN (NeuN+) cell bodies were counted and stereotaxically mapped to the Allen Brain Atlas (Allen, 2004). We found that along the entire length of the A-P axis, six major brain areas [lateral septal nucleus (LS), bed nuclei of the stria terminalis (BST), dorsal striatum (STRd), amygdala (Amyg), hypothalamus (HY), and the ventral periaqueductal gray (vPAG)] contained Nav1.8+ cell bodies in adult (10–12 weeks old) female or male Nav1.8-Cre-tdT mice (Figure 1). Notably, we found no or almost no Nav1.8+ cell bodies in the cerebral cortex, hippocampal formation, thalamus, cerebellum, or spinal cord. In the LS, we observed Nav1.8+ cells along the entire length of the A-P axis of the rostral (LSr), caudal (LSc), and ventral (LSv) parts, but with an increased proportion in the anterior regions of LSr (7.13 ± 1.38% at 1.04 mm) and LSv (8.75 ± 0.85% at 0.14 mm) (Figures 1A–G,Q–S). In the BST, we found that 3.16 ± 0.70% of Nav1.8+ cell bodies are present in the region 0.08 mm posterior to bregma, whereas <1% (0.72 ± 0.20%) are located more anteriorly (0.62 mm) (Figures 1F,H,T). The STRd has the highest absolute number of Nav1.8+ cell bodies. These cells are particularly dense in the medial portion of the posterior STRd (−1.55 mm) and noticeably less dense in the lateral zone close to the corpus callosum (Figures 1K,U). Therefore, Nav1.8+ density in STRd is only 2.60 ± 0.52% at −1.55 mm, as a result of the analysis considering the whole area. We also observed Nav1.8+ cell bodies in both the central (CEA) and the three major clusters of the medial (MEA) amygdalar nuclei (Figures 1L,V). Most of these cells are located in the lateral part of CEA (CEAl) (5.92 ± 0.70% at −1.55 mm), where they appear to be crowded together. Within the hypothalamus, Nav1.8+ cell bodies are found predominantly in the medial (mHY) and anterior lateral (LHA) areas, where they achieve up to 5.71 ± 1.52% at LHA −0.48 mm (Figures 1I,J,M–O,W,X). Nav1.8+ cell bodies are located only in the anterior vPAG (3.59 ± 0.28% at −2.55 mm), where they are distributed more ventrally between the wall of the aqueduct and the fasciculus retroflexus (Figures 1P,Y). Overall, our density mapping revealed that Nav1.8+ cell bodies are more predominant within the PAG and cerebral nuclei, particularly in LSv, LSr, and amygdala (Supplementary Figure S1, Table S3).

### Perisomatic morphology of Nav1.8 neurons

**Figure 2:**
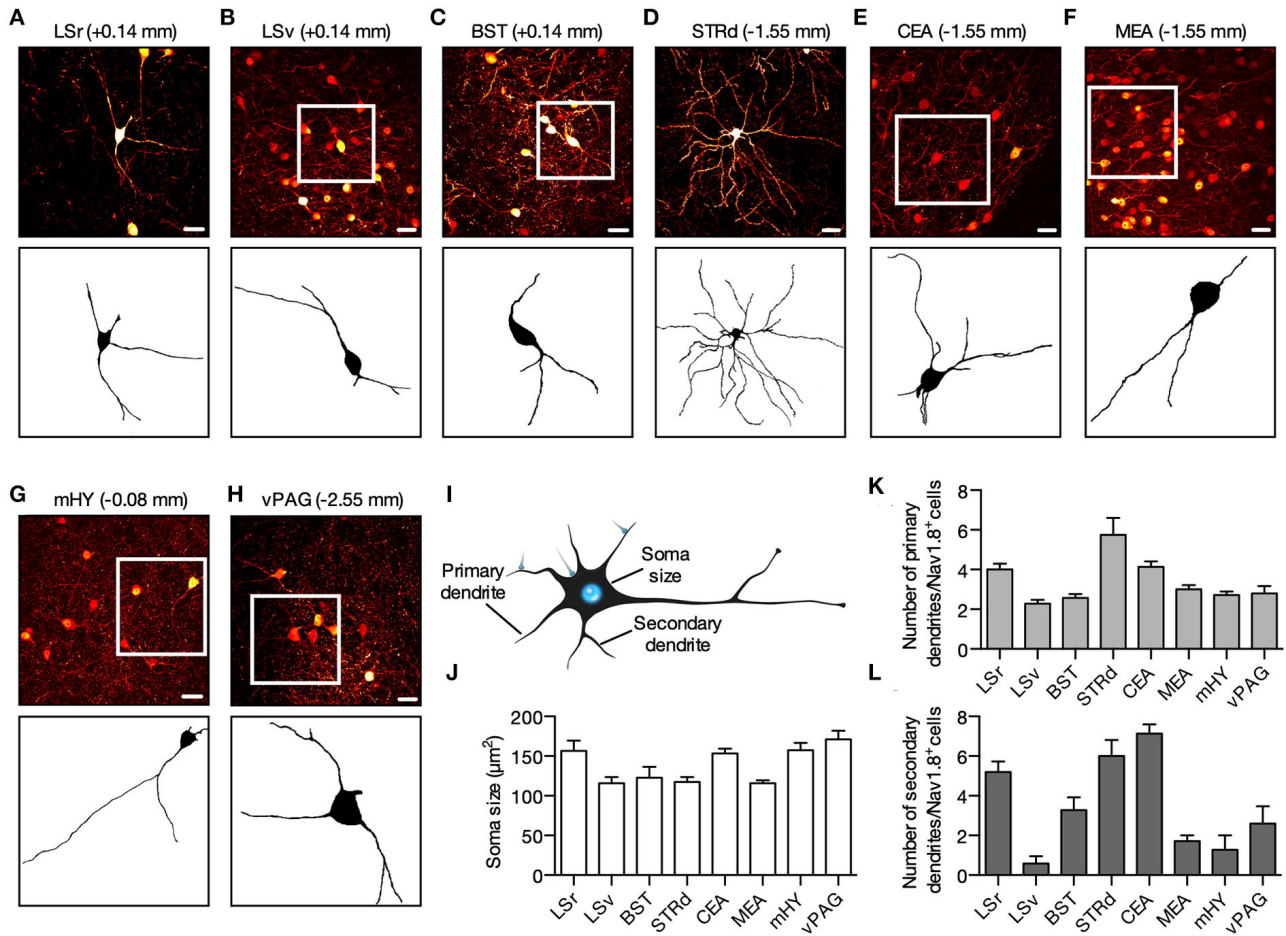
Perisomatic morphology of Nav1.8 neurons. **(A–H)** Representative maximum projection coronal sections (top) and binary transformation (bottom) to show the perisomatic morphological features of Nav1.8+ neurons from Nav1.8-Cre-tdT mice. Fluorescence is pseudo-colored in red hot to reflect differences in intensity. **(I)** The morphometric study measured the soma size (cross-sectional area), the number of primaries (branch emerging from the soma), and secondaries (branch emerging from a primary dendrite) dendrites. **(J–L)** The graphs show the quantification of the soma size **(J)**, and the number of primaries **(K)** and secondaries **(L)** dendrites (*n* = 3 male mice, 10**–**20 neurons per animal). BST, bed nuclei of the stria terminalis; CEA, central amygdalar nucleus; LSr and LSv, rostral and ventral part of the lateral septal nucleus; mHY, medial hypothalamus; vPAG, ventral periaqueductal gray; STRd, striatum dorsal region. Scale bar = 25 μm. Summary data are represented as mean ± SEM. The drawing in this figure **(I)** is in the public domain and available in http://pngaaa.com.

Confocal analysis revealed that Nav1.8 neurons possessed significative perisomatic morphological differences across the brain areas studied. Our morphometric study measured the soma size and the number of primary (branch emerging from the soma) and secondary (branch emerging from a primary dendrite) dendrites (Figure 2I). Within LS, most Nav1.8 neurons are fusiform (Figures 2A,B). These neurons are distributed sparsely and their soma size in LSr and LSv is 156.5 ± 13.17 and 116.0 ± 7.59 μm^2^, respectively (Figures 2A,B,J). Nav1.8 neurons in these regions are bipolar since the pattern of dendritic arborization revealed that they often display two main dendrites oriented in opposite directions. Although the extent of the proximal ramification analysis using thin histologic sections (100 μm) should be interpreted cautiously, we found that within LS, the Nav1.8 neurons from LSr display greater dendritic arborization than those from LSv (primary dendrites: LSr = 4.0 ± 0.29 vs. LSv = 2.0 ± 0.18; secondary dendrites: LSr = 2.0 ± 0.5 vs. LSv = 0.57 ± 0.3; Figures 2K,L). Typically, LS Nav1.8 neurons displayed fine caliber axons that followed a dorsoventral trajectory toward the hypothalamus. Overall, Nav1.8 neurons in BST and LS seem identical. BST neurons also present a fusiform shape and medium-sized soma (soma size 122.9 ± 13.60 μm^2^; primary dendrites 2.6 ± 0.20; secondary dendrites 3.3 ± 0.6; Figures 2C,J–L). We observed that the BST Nav1.8 neurons displayed the highest tdT fluoresce in the brain, as suggested by the differences in intensity from pseudo-colored images. In the STRd, Nav1.8 neurons are multipolar with more than 3 dendrites radiating in all directions (Figure 2D). Their mean somatic size is 117.4 ± 6.19 μm^2^, which is among the smallest found in the areas investigated (including 116.0 ± 7.59 μm^2^ for the LSv and 115.9 ± 3.69 μm^2^ from MEA; Figures 2B,D,F,J). However, dendritic arborization of Nav1.8 neurons is significantly more profuse in STRd than in other brain areas, as judged by the increased number of primary and secondary dendrites (primary dendrites: 5.75 ± 0.85; secondary dendrites: 6.0 ± 0.7; Figures 2K,L). Whereas Nav1.8 neurons were rare and randomly distributed in the dorsolateral and ventrolateral subdivisions, these neurons tended to accumulate preferentially in the most medial STRd subdivision close to the pallidum. As these STRd Nav1.8 neurons display medium-sized soma and extensive dendritic trees containing stubby spines, we believe that they are spiny projection neurons (SPNs) [also known as medium spiny neurons (MSNs)] as indicated by our 3D reconstruction of the confocal scanning (Supplementary Movie S1). The majority of Nav1.8 neurons in the amygdala (CEA and MEA), mHY, and vPAG are pyramidal, as they often show one apical and two basal dendrites (Figures 2E–H). We also perceived that the mHY Nav1.8 neurons are also spherical or fusiform and usually exhibit two or three primary dendrites (Figure 2G). Soma size of Nav1.8 neurons was 157.4 ± 9.30 and 1,71.2 ± 10.91 μm^2^ for mHY and vPAG, respectively (Figures 2G,H,J). The average number of primary dendrites is fairly similar among Nav1.8 neurons in CEA, MEA, mHY, and vPAG (Figures 2K,L). Nevertheless, the junction between the primary and secondary dendrites within mHY and vPAG occurred much farther from the cell body (Figures 2G,H). The CEA Nav1.8 neurons present numerous sparse dendrite spines and the highest number of secondary dendrites across the brain areas investigated (7.14 ± 0.45; Figure 2L). The increased somatodendritic domain of Nav1.8 neurons in CEA makes a remarkable ramification overlapping within this region (Figure 1E).

### Neurotransmitter phenotype of Nav1.8 neurons in the brain

**Figure 3:**
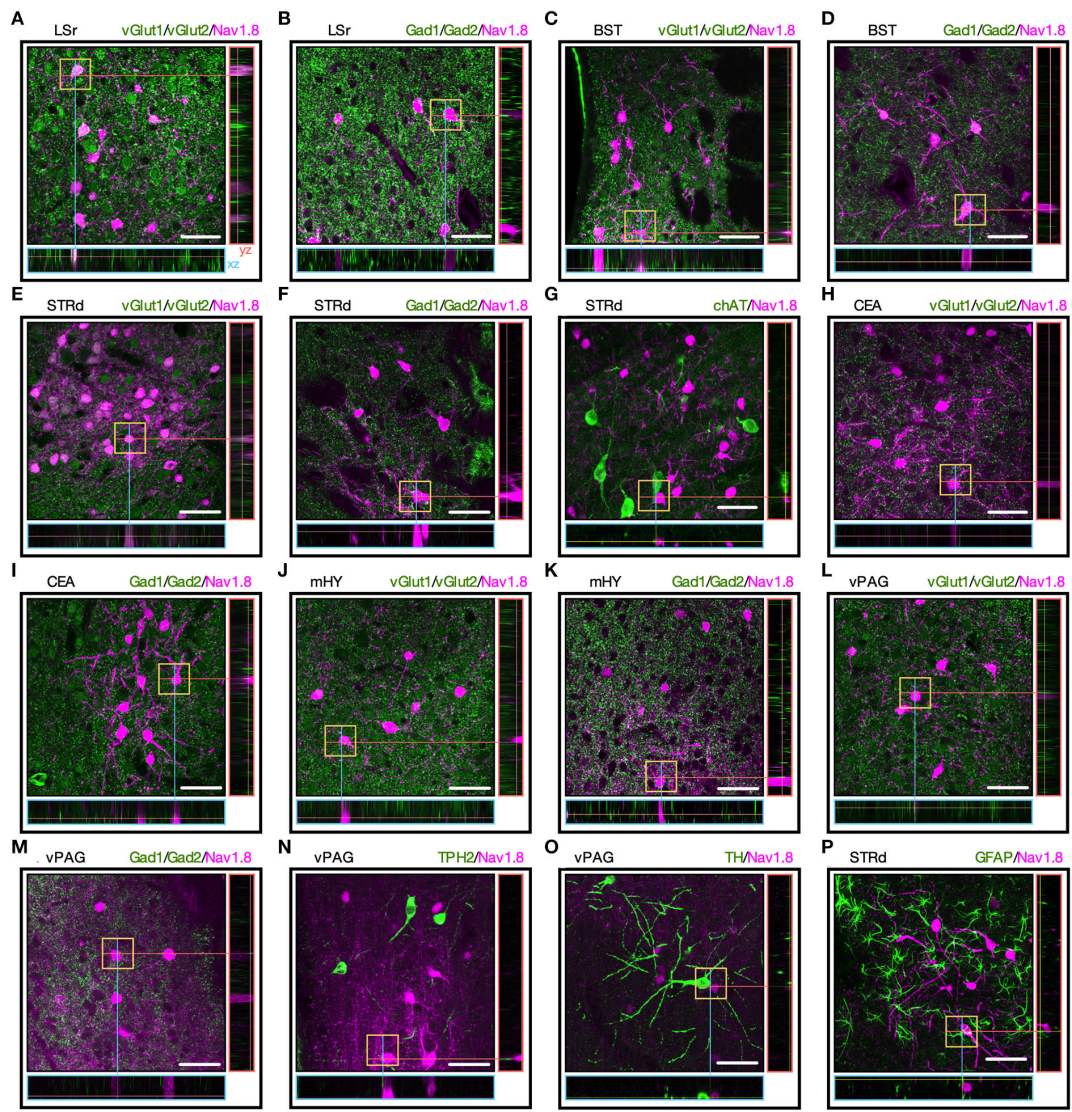
The neurotransmitter phenotype of Nav1.8 neurons. **(A–O)** Representative maximum projection coronal sections with orthogonal views (xz and yz) containing tdT fluorescence (magenta) and immunoreactivity for the neurotransmitter markers (green) in Nav1.8-Cre-tdT mice. vGlut1/2 (vesicular glutamate transporter; for glutamatergic neurons), Gad1/2 (glutamate decarboxylase 1/2, for GABAergic neurons), ChAT (choline acetyltransferase, for cholinergic neurons), TPH2 (tryptophan hydroxylase 2, for serotoninergic neurons) or TH (tyrosine hydroxylase, for dopaminergic or noradrenergic neurons). **(P)** Colocalization of tdT with the astrocyte marker GFAP (green). Double-labeled cell bodies are white (*n* = 3 male mice). BST, bed nuclei of the stria terminalis; CEA, central amygdalar nucleus; LS, lateral septal nucleus; LSr, rostral part of the lateral septal nucleus; mHY, medial hypothalamus; PAG, periaqueductal gray; STRd, striatum dorsal region. Scale bar = μm.

Since the neurotransmitter phenotype of Nav1.8 neurons in the brain is uncertain, we mapped Nav1.8+ neurons colabeled with antibodies targeting the major brain neurotransmitter cell types, including Gad1/2 (for GABAergic), vGlut1/2 (for glutamatergic), ChAT (for cholinergic), TPH2 (for serotonergic), or TH (for dopaminergic or noradrenergic). We incubated these antibodies with selected brain slices where Nav1.8 neurons are more densely clustered and according to the expression pattern reported in the Allen Brain Atlas database (Allen, 2004). We found a high degree of co-localization between Nav1.8^+^ with vGlut1/2 or Gad1/2 in different brain areas. While the Nav1.8 neurons from LSr are glutamatergic (Figures 3A,B), those from STRd and CEA are GABAergic as tdT colocalized with the Gad1/2 marker (Figures 3E–I). Nav1.8 neurons from BST were colabeled with both vGlut1/2 and Gad1/2 (Figures 3C,D). Furthermore, no colabeling between Nav1.8+ with any neurotransmitter markers was observed in mHY and PAG (Figures 3J–O).Finally, we also did not find any colabeling between Nav1.8^+^ and the glial markers GFAP (Figure 3P) and Iba1 (Supplementary Figure S2), thus suggesting that Nav1.8 is not expressed in non-neuronal cells within the brain.

### Nav1.8 promoter is activated transiently in the brain

**Figure 4:**
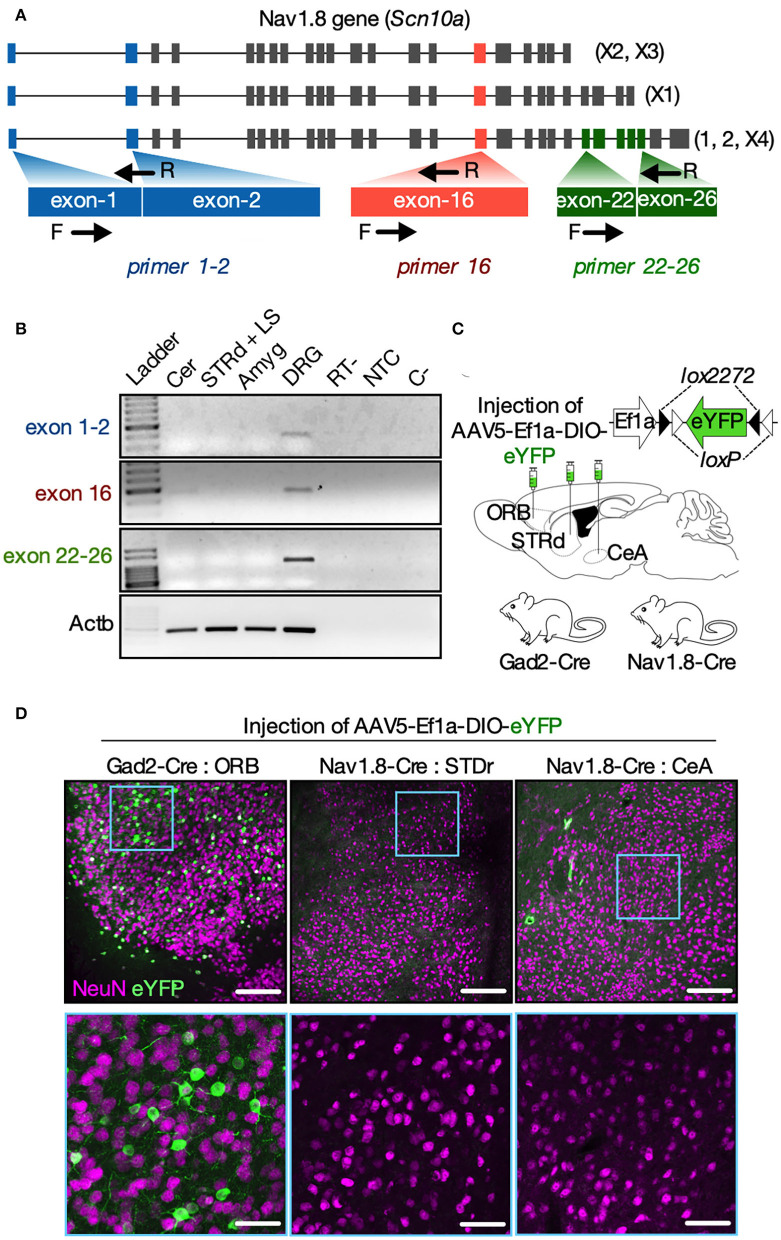
Nav1.8 (*Scn10a*) gene expression is inactive in the brain of adult mice. **(A)** Mouse Nav1.8 (*Scn10a*, gene ID 20264) transcript isoforms and a schematic of the three primer sets used: primers exon1-2 (blue) recognize either the inclusion of exon 1 or the exon 1-2 junction (in all isoforms); primers 16 (red) recognize the exon 16 (in all isoforms); primers exon 22**–**26 (green) recognize a region in exon 26 unique to the isoforms 1, 2 and X4. **(B)** Representative agarose gel electrophoresis from the RT-PCR analysis for Nav1.8 (exon 1**–**2; 16 and 22**–**26) or beta-actin (Actb, housekeeping) in the cerebellum (Cer), dorsal striatum plus septum (STRd + LS), amygdala (Amyg) or dorsal root ganglia (DRG). Experimental controls RT- (cDNA synthesis without reverse transcriptase), NTC (cDNA synthesis without RNA), and C- (PCR without cDNA) are also shown (n = 5 male mice). **(C)** The experimental approach to deliver an AAV carrying a Cre-dependent eYFP reporter (AAV-Ef1a-DIO-eYFP) into the cortical orbital area (ORB, Gad2-cre mice only), dorsal striatum (STRd) or central amygdala (CeA) of Gad2-Cre or Nav1.8-Cre mice (*n* = 2**–**3 male mice/group). **(D)** Low (top panels, scale bar = 150 μm) or high (bottom panels, scale bar = 50 μm) magnification coronal sections displaying EYFP-positive neurons (green) co-stained with the neuronal marker NeuN (magenta) at the injection site. EYFP-positive neurons were detected only in the ORB of Gad2-Cre mice (on the left). The drawings in **(A,C)** were created with the Keynote software version 9.2.1 (http://apple.com/keynote).

Since tdT fluorescence in the brain of Nav1.8-Cre-tdT mice results from the *Scn10a* gene promoter activity at any time during development, the following experiment investigated whether there is *Scn10a* gene expression in the brain regions of adult mice carrying Nav1.8+ neurons. We microdissected the striatum (containing the septal area), amygdala, cerebellum, and the DRG from fresh tissue slices obtained from WT mice at postnatal day 57 (8 weeks old). These samples were subjected to reverse transcription polymerase chain reaction (RT-PCR) experiments using three primer sets designed to detect all known *Scn10a* transcripts (isoforms 1, 2, X1, X2, X3, and X4) (Figure 4A). Although all the primer sets could detect the *Scn10a* mRNA in the DRG, no transcript isoforms were expressed in the brain regions investigated (Figure 4B). These findings, however, are premature to exclude the expression of the *Scn10a* gene in the adult mouse brain, in part because the reliability of the RT-PCR to detect transcriptional activity is affected by post-transcriptional gene silencing, which could induce a rapid degradation of mRNA (Bevilacqua et al., 2003). Thus, we also used an AVV approach to re-examine the *Scn10a* promoter activity in adult mice (Figure 4C). We injected an AAV carrying a Cre-dependent eYFP reporter (AAV5-Ef1a-DIO-eYFP) into the striatum and amygdala of Nav1.8-Cre mice to evaluate the Cre recombinase activity and therefore *Scn10a* promoter activity. As an experimental control, we also injected AAV5-Ef1a-DIO-eYFP into the orbital cortex (ORB) of Gad2-Cre mice, which show robust Cre recombinase activity throughout the cortex, striatum, and cerebellum as reported elsewhere (Allen Brain Atlas experiment #100117967). Then, 3 weeks later, fluorescence microscopy analysis revealed that viral injection resulted in strong expression of eYFP in cortical neurons of Gad2-Cre mice as expected (Figure 4D; left panel). In contrast, no virus-transduced neurons were detectable in both the striatum and amygdala of Nav1.8-Cre mice (Figure 4D; middle and right panels).

Since the *Scn10a* promoter appears to be inactive in adult mice and Cre-mediated recombination permanently activates tdT expression, our findings suggest that Nav1.8 is expressed transiently in the brain, likely during prenatal development.

### Chronic pain increases the activity of Nav1.8 neurons

Inflammatory mediators known to produce hyperalgesia can activate nociceptor terminals of sensory Nav1.8 neurons from the dorsal root ganglia (DRG) (Hudmon et al., 2008; Chiu et al., 2013). Hyperalgesia triggered by noxious stimuli is attenuated in Nav1.8-knockout mice (Akopian et al., 1999; Kerr et al., 2001; Laird et al., 2002) and mice treated with Nav1.8 channel blocker (Jarvis et al., 2007) or antisense oligonucleotides (Khasar et al., 1998; Porreca et al., 1999; Yoshimura et al., 2001). Whereas Nav1.8 has been well studied in the peripheral nervous system, its pathophysiological role in brain neural networks is largely ignored as this ion channel is thought to be absent in the central nervous system. Given that Nav1.8 appears to be transiently expressed in major limbic structures believed to mediate the emotional component of pain (Kuner, 2010), we sought to understand whether transiently Nav1.8-expressing neurons are capable of sensing noxious stimuli in the brain.

**Figure 5:**
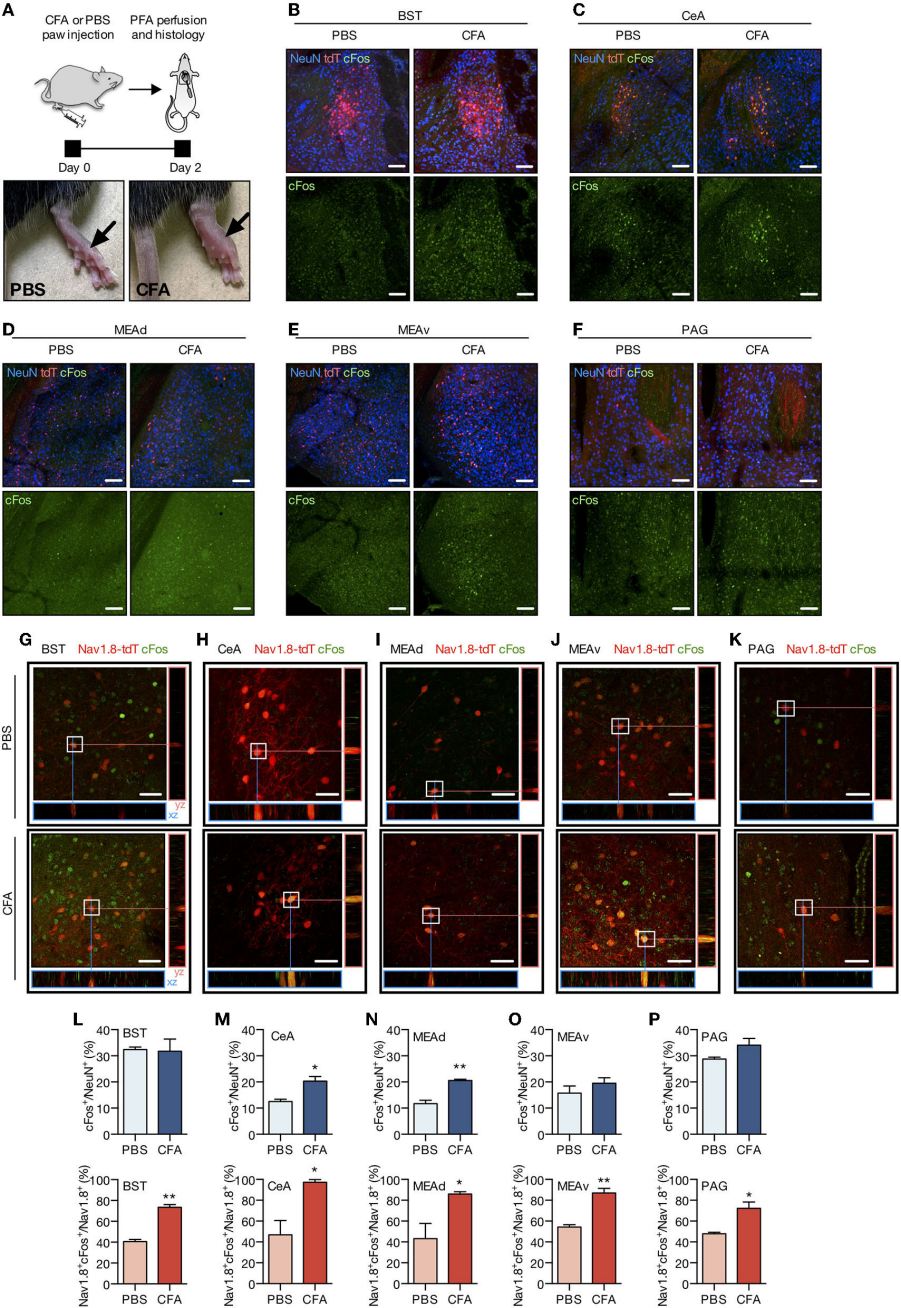
Chronic pain increases the activity of Nav1.8 neurons. **(A)** Experimental design for the CFA (complete Freund's adjuvant) model of chronic inflammatory pain. CFA or PBS were injected into both hind paws of Nav1.8-Cre-tdT mice. Two days later, the brain was collected for the histology studies. The arrow on the right side shows an inflamed paw from a CFA-injected mouse compared to the PBS-injected mouse on the left (*n* = 3 male mice). **(B–F)** Representative fluorescent images of NeuN-, tdT- and cFos-labeled neuronal bodies in the PBS and CFA groups 2 days after the paw injection. **(G–K)** Representative maximum projection coronal sections with orthogonal views (xz and yz) containing tdT fluorescence (red) and immunoreactivity for cFos (green) in Nav1.8-Cre-tdT mice injected with PBS (top) or CFA (bottom). Double-labeled cell bodies are yellow. **(L–P)** Density of cFos+ neuronal bodies relative to NeuN+ cells (cFos+/NeuN+ on the top) or double-labeled Nav1.8+/cFos+ neuronal bodies relative to the total Nav1.8+ neurons (Nav1.8+cFos+/Nav1.8+, on the bottom). All images are from the right hemisphere. BST: bed nuclei of the stria terminalis; CEA: central amygdalar nucleus; MEAad and MEAav: medial amygdalar nucleus, anterodorsal (ad) or anteroventral (av) part; PAG: periaqueductal gray. Scale bar = 40 μm. Summary data are represented as mean ± SEM. Significant differences were determined by Student's *t*-tests. Asterisks (*) in the figures indicate the *p*-values for the *post-hoc* test and correspond to the following values: *p < 0.05; **p < 0.01. The drawings in **(A)** were created with the Keynote software version 9.2.1 (http://apple.com/keynote).

To this regard, we injected the complete Freund's adjuvant (CFA) or PBS into both hind paws of adult Nav1.8-Cre-tdT mice (10 μl per animal) to recapitulate a model of chronic inflammatory pain (Figure 5A). Our previous study shows that this experimental model displays mechanical allodynia for up to 14 days (Tenza-Ferrer et al., 2019). Conversely, injection of vehicle does not change the mechanical nociceptive threshold. Then 2 days after injection, brain tissue was collected to study the expression of the immediate-early gene cFOS as a proxy of neuronal activity (Kovács, 2008; Magno et al., 2019). Changes in the expression of cFOS were largely related to chronic inflammatory pain as the CFA-injected mice exhibited increased density of cFOS/NeuN double-positive neurons (cFos+/NeuN+) in CeA (Figures 5C,M; PBS: 12.54 ± 0.84%; CFA: 20.34 ± 1.75%; mean difference (MD) = 7.79, 95% CI [2.41–13.18], t(4) = 4.02, *p* = 0.0159, *n* = 4/group, Student's *t*-test) and MEAd (Figures 5D,N; PBS: 11.74 ± 1.29%; CFA: 20.57 ± 0.50%; MD = 8.83, 95% CI [4.98–12.68], t(4) = 6.36, *p* = 0.0031, *n* = 4/group, Student's *t*-test) compared to the PBS-injected group. CFA and PBS groups showed similar distribution of cFos+/NeuN+ in BST, MEAv, and PAG (statistics for BST: PBS: 32.38 ± 1.01%; CFA: 31.72 ± 4.66%; MD = −0.6567, 95% CI [−19.94–18.63], t(4) = 0.1084, *p* = 0.9205; statistics for MEAv: PBS: 15.71 ± 2.75%; CFA: 19.54 ± 2.10%; MD = 3.82, 95% CI [−5.78–13.43], t(4) = 1.10, *p* = 0.3311; statistics for PAG: PBS: 28.79 ± 0.69%; CFA: 34.06 ± 2.63%; MD = 5.28, 95% CI [−2.18–12.83], t(4) = 1.94, *p* = 0.1246; in all cases n = 4/group, Student's *t*-test; Figures 5B,E,F,L,O,P). We also assessed co-localization between c-Fos and tdT to investigate whether transiently Nav1.8-expressing neurons are activated following CFA administration. Indeed, transiently Nav1.8-expressing neurons were remarkably activated since the majority (between 70 and 97%) of these neurons co-expressed cFOS in the CFA-injected group compared to the PBS group (between 40 and 55%). Notably, Nav1.8+/cFOS+ neurons were enriched in all regions investigated of mice with chronic inflammatory pain (statistics for BST: PBS: 40.60 ± 2.02%; CFA: 73.50 ± 2.61%; MD = 32.91, 95% CI [21.17–44.6], t(4) = 8.92, *p* = 0.0030; statistics for CeA: PBS: 46.86 ± 13.68%; CFA: 97.22 ± 2.78%; MD = 50.37, 95% CI [11.60–89.13], t(4) = 3.60, *p* = 0.0226; statistics for MEAd: PBS: 43.17 ± 14.67%; CFA: 85.95 ± 2.35%;MD = 42.78 95% CI [1.53–84.03], t(4) = 2.88, *p* = 0.0450; statistics for MEAv: PBS: 54.38 ± 2.2%; CFA: 87.10 ± 4.41%; MD = 32.72, 95% CI [19.01–46.42], t(4) = 6.63, *p* = 0.0027; statistics for PAG: PBS: 47.91 ± 1.32%; CFA: 72.32 ± 6.02%; MD = 24.41, 95% CI [7.30–41.53], t(4) = 3.96, *p* = 0.0167; in all cases n = 4/group, Student's *t*-test; Figures 5G–P).

Overall, these findings suggest that transiently Nav1.8-expressing neurons may drive brain activation following the chronic noxious stimulus.

## Discussion

Primary sensory neurons from the dorsal root ganglion (DRG) that express the Nav1.8 sodium channel have been extensively investigated, mainly for their role in generating neuronal hyperexcitability in pain-related conditions (Black et al., 2004; Zimmermann et al., 2007; Hameed, 2019; Goodwin and McMahon, 2021). In contrast, less is known regarding the role of encephalic Nav1.8 neurons. In this study, we have provided a comprehensive approach to mapping the precise locations of transiently Nav1.8-expressing neurons within the brain of a Nav1.8-Cre mouse line. Moreover, we provided the first demonstration that transiently Nav1.8-expressing neurons are capable of sensing noxious stimuli in the brain.

Since we found no mRNA expression nor promoter activity of the Nav1.8 gene (*Scn10a*) within the investigated brain areas from adult mice (Figure 4), we believe that tdT fluorescence in the brain of Nav1.8-Cre-tdT mice might be triggered by the activation of the *Scn10a* promoter during the prenatal development. This finding is in line with the prior results showing that Nav1.8 is expressed during embryogenesis, although its protein is absent in the central nervous system of adult mice (Benn et al., 2001; Stirling et al., 2005; Gautron et al., 2011). Thus, the brain regions of the adult mice carrying tdT+ neuronal bodies (Figure 1) may reflect the neuronal subpopulations that expressed Nav1.8 transiently in the developing brain. Indeed, evidence has shown that several genes coding for ionic channels change their expression pattern in the developing brain, with transcript levels falling strongly in the early postnatal periods or even the contrary (Beckh et al., 1989; Bettler et al., 1990; Monyer et al., 1994; Waxman et al., 1994; Benn et al., 2001; Staaf et al., 2010). Thus, it is likely that Nav1.8 expression within the brain occurs prenatally and therefore Nav1.8 neuronal subpopulations may influence neural development, which is in accordance with the earlier reports (Benn et al., 2001). Nevertheless, the mechanism of how Nav1.8 influences brain development or when the protein expression is disrupted in the brain shortly before birth is yet to be investigated.

On the other hand, the distinct temporal expression pattern of Nav1.8 during development would mean that the Nav1.8 neuronal subpopulations across the nervous system might display functional heterogeneity. The findings of our study are in line with this. First, transiently Nav1.8-expressing neurons are located in different brain areas, including the lateral septal nucleus (LS), bed nuclei of the stria terminalis (BST), dorsal striatum (STRd), amygdala (Amyg), hypothalamus (HY), and the ventral periaqueductal gray (vPAG). Thus, the precise locations revealed by our whole-brain map approach provide a step toward establishing the neurophysiological role of diverse Nav1.8 subpopulations.

Second, Nav1.8 neurons exhibit distinct perisomatic morphology across the brain areas studied, further reinforcing that Nav1.8 neurons in the brain are distinct subpopulations. For example, within the anterior striatum (LS and BST), most Nav1.8 neurons are bipolar with few dendrites branching occasionally. These neurons were found evenly dispersed throughout these nuclei. More posteriorly, Nav1.8 neurons within the STRd exhibit a higher morphological complexity and could be divided into two distinct populations: one located in the internal part close to the globus pallidum containing densely packed neurons; and the second, formed as a band of neurons circumventing the lateral part of the STRd. The STRd Nav1.8 neurons are remarkably distinct from that of anterior brain areas as they are multipolar and display a profuse dendritic arborization that resembles spiny projection neurons, which are the main source of striatal GABA-projecting neurons.

Third, the Nav1.8 neuronal populations in the brain can also be grouped into different niches according to their neurochemical identity. For example, we demonstrated for the first time that almost every neuron in STRd is immunoreactive to Gad1/Gad2 markers, suggesting that Nav1.8 neurons within the STRd (1) may release GABA and (2) therefore exert GABA-mediated monosynaptic transmission to regulate the basal nuclei function *via* striatal outputs. Thus, striatal Nav1.8 neurons could integrate afferent signals from the cortex or substantia nigra during motor behaviors (Sano et al., 2013), which highlights the need for future research to determine whether these cells are interneurons or projection neurons. Although most Nav1.8 neurons are also GABAergic in the amygdala, we found that across LSr, Nav1.8 neurons are glutamatergic, whereas the populations from mHY and PAG did not co-express any neurotransmitter markers tested, such as Gad1/2 (for GABAergic), vGlut1/2 (for glutamatergic), chAT (for cholinergic), TPH2 (for serotonergic), or TH (for dopaminergic or noradrenergic). Furthermore, we highlight that molecular identification of Nav1.8 neurons could be improved through fluorescence-activated cell sorting (FACS), which would allow the isolation of tdT-positive neurons from the sorted pool and the transcriptome analysis of neural markers (Yelin-Bekerman et al., 2015).

Activation of sensory Nav1.8 neurons from the PNS produces hyperalgesia (Daou et al., 2016). This poses the question: Are the populations of Nav1.8 in the brain also involved in the pain neurotransmission? This is interesting since all the regions where we have found the transiently Nav1.8-expressing neurons are located within the limbic circuitry, a key mediator of the emotional component of pain. In this regard, we measured the activity of Nav1.8 neurons through the expression of the immediate-early gene cFOS in an experimental model of pain. We are the first to show that the transiently Nav1.8-expressing neurons from BST, amygdala, and PAG are capable of sensing noxious stimuli in the brain, as the number of these neurons that co-expressed cFOS was higher in the experimental model of the pain group compared to the sham group. The CEAl receives nociceptive neurotransmission from the spinal cord and the brainstem through the spinoparabrachial amygdaloid pathway (Bourgeais et al., 2001). Similarly, the BST also receives direct and indirect nociceptive inputs from the spinal dorsal horn and limbic regions (including the amygdala and ventromedial hypothalamus), which is thought to exert an important role in the negative-emotional component of pain (Gauriau and Bernard, 2002; Braz et al., 2005; Minami, 2019). Moreover, the PAG has several nuclei involved in the processing of nociceptive inputs from the spinal cord that shape the experience of pain (Neugebauer et al., 2004; Rodríguez-Muñoz et al., 2012; Eippert and Tracey, 2014).

Finally, the new involvement of transiently Nav1.8-expressing neurons from the brain with pain conditions may have therapeutic implications. It would be interesting to evaluate whether the optogenetic inhibition of Nav1.8 brain subpopulations could attenuate pain in animal models and demonstrate the specific role of these neurons in different pain contexts. Nevertheless, our study could reveal the distribution of these transiently Nav1.8-expressing neurons in the whole brain, and therefore, these findings will be facilitating the comprehension of discoveries linking Nav1.8 and pain.

## Data availability statement

The original contributions presented in the study are included in the article/Supplementary material, further inquiries can be directed to the corresponding authors.

## Ethics statement

The animal study was reviewed and approved by Comité de ética no uso de animais (CEUA) - (protocol 280/2019) - Federal University of Minas Gerais, Belo Horizonte, Brazil.

## Author contributions

HT-F, MC, EN, and LM conducted all the experiments and the analyses. HT-F and LM conceived and designed the experiments and wrote the manuscript. AB and MR-S reviewed the manuscript. All authors contributed to the article and approved the submitted version.

## Funding

This study was supported by the grants from Conselho Nacional de Desenvolvimento Cientifico e Tecnológico (CNPq) (457639/2014-8) for LM, Coordenação de Aperfeiçoamento de Pessoal de Nível Superior – Brasil (CAPES) – Finance Code 001 for HT-F and LM, Fundação de Amparo à Pesquisa do Estado de Minas Gerais - FAPEMIG (APQ 00476-14) for MR-S, and Instituto Serrapilheira/Serra-1708-15285 and FAPEMIG (APQ-01321-21; Rede Mineira de Pesquisa Translacional em Imunobiológicos e Biofármacos no Câncer [REMITRIBIC, RED-00031-21]; Rede Mineira de Engenharia de Tecidos e Terapia Celular [REMETTEC, RED-00570-16]) for AB. MR-S and AB are supported by a research productivity fellowship from CNPq.

## Conflict of interest

The authors declare that the research was conducted in the absence of any commercial or financial relationships that could be construed as a potential conflict of interest.

## Publisher's note

All claims expressed in this article are solely those of the authors and do not necessarily represent those of their affiliated organizations, or those of the publisher, the editors and the reviewers. Any product that may be evaluated in this article, or claim that may be made by its manufacturer, is not guaranteed or endorsed by the publisher.

## Supplementary material

Supplementary Figure S1 Nav1.8 population. Diagrammatic representation of Nav1.8 population all over the brain. Amyg, amygdalar nuclei; ACB, nucleus accumbens; BST, bed nuclei of the stria terminalis; CTX, cerebral cortex; HY, hypothalamus; LSr, rostral part of the lateral septal nucleus; LSv, ventral part of the lateral septal nucleus; PAG, periaqueductal gray; STRd, striatum dorsal region; TH, thalamus. The drawings were created with the Keynote version 9.2.1 and Fiji software.

Supplementary Figure S2 tdT is absent in microglia. Representative maximum projection coronal sections with orthogonal views (xz and yz) containing tdT fluorescence (magenta) and immunoreactivity for the microglial marker Iba1 (green). STRd, striatum dorsal region. Scale bar = 25 μm.

Supplementary Movie S1 The 3D reconstruction of a striatal Nav1.8 neuron.

Supplementary Table S1 The sequences of the primer sets used in the study.

Supplementary Table S2 The *Mus musculus Scn10a* gene (gene ID 20264) transcripts.

Supplementary Table S3 Brain regions and respective anterior-posterior coordinates (in mm) where Nav1.8 neurons were quantified.
