# Supplementary figures and images for: Transiently Nav1.8-expressing neurons are capable of sensing noxious stimuli in the brain
Source: Front Cell Neurosci. 2022 Aug 29;16:933874. doi: 10.3389/fncel.2022.933874 (PMC9464809; doi:10.3389/fncel.2022.933874)

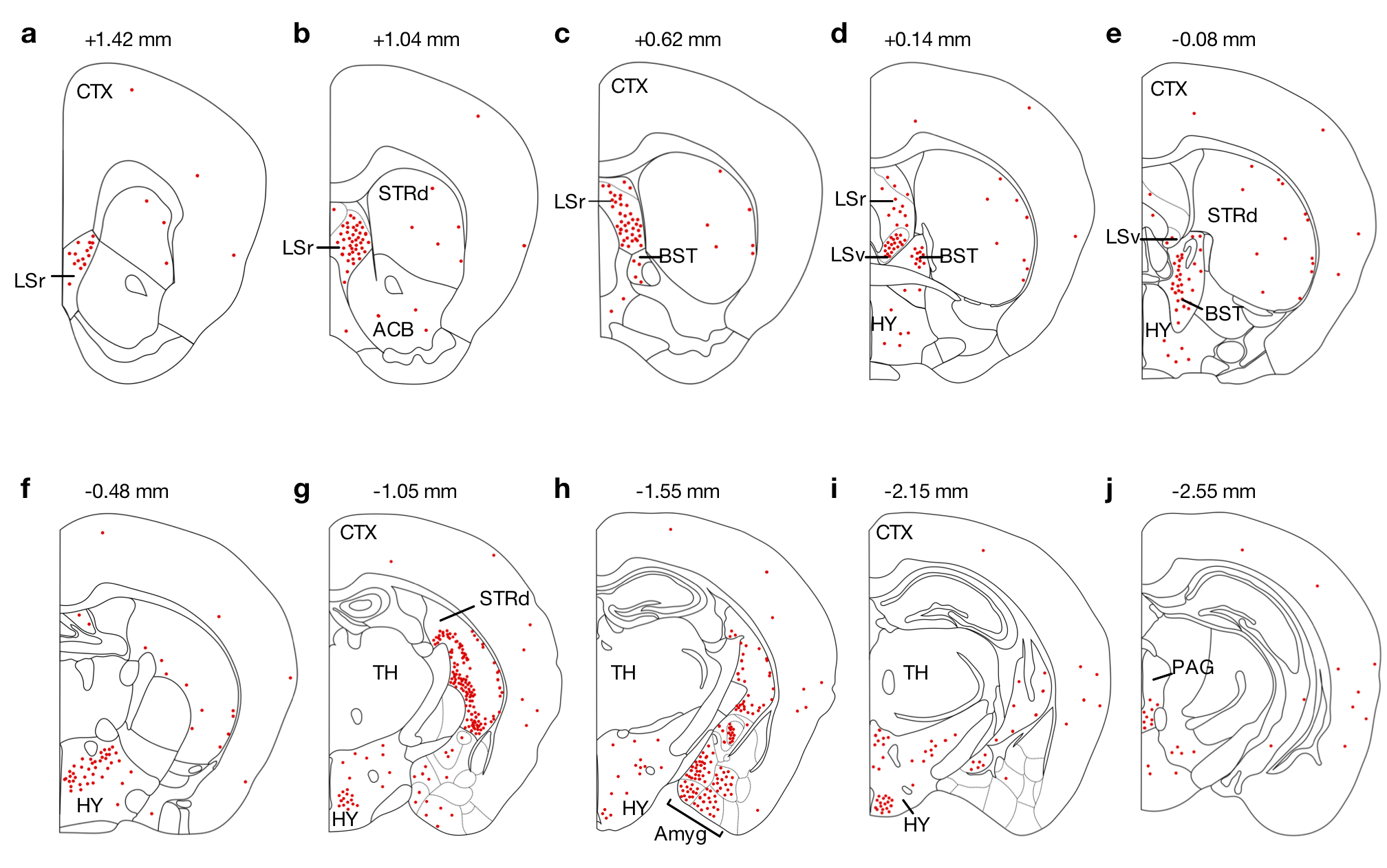

Supplement: Supplementary Figure S1 — Nav1.8 population. Diagrammatic representation of Nav1.8 population all over the brain. Amyg, amygdalar nuclei; ACB, nucleus accumbens; BST, bed nuclei of the stria terminalis; CTX, cerebral cortex; HY, hypothalamus; LSr, rostral part of the lateral septal nucleus; LSv, ventral part of the lateral septal nucleus; PAG, periaqueductal gray; STRd, striatum dorsal region; TH, thalamus. The drawings were created with the Keynote version 9.2.1 and Fiji software. [file Image_1.TIF]
