# Supplementary material for: Transiently Nav1.8-expressing neurons are capable of sensing noxious stimuli in the brain
Source: Front Cell Neurosci. 2022 Aug 29;16:933874. doi: 10.3389/fncel.2022.933874 (PMC9464809; doi:10.3389/fncel.2022.933874)
Supplement: Supplementary Table S1 — The sequences of the primer sets used in the study. [file Table_1.DOCX]

**Supplementary Table 1.** The sequences of the primer sets used in the study.

| **Primer** | **Forward** | **Reverse** |
| --- | --- | --- |
| Nav1.8-exon1_2 | CCCCGGACAAGTAAGAGTGT | GGGCTTCTCACTCTTGTCCT |
| Nav1.8-exon16 | TCCTGGCCATCATCGTCTTT | CAAGAAGAGGGTGAGGCAGA |
| Nav1.8-exon22_26 | GTCCATCAAAGCCCTTCGGA | TCTGTCATGAAGATGTCCTGGC |
