# Supplementary material for: Transiently Nav1.8-expressing neurons are capable of sensing noxious stimuli in the brain
Source: Front Cell Neurosci. 2022 Aug 29;16:933874. doi: 10.3389/fncel.2022.933874 (PMC9464809; doi:10.3389/fncel.2022.933874)
Supplement: Supplementary Table S2 — The Mus musculus Scn10a gene (gene ID 20264) transcripts. [file Table_2.DOCX]

**Supplementary Table 2.** The *Mus musculus* *Scn10a* gene (gene ID 20264) transcripts

| **Isoform** | **Transcript** | **Length (nt)** | **Protein** | **Length (aa)** |
| --- | --- | --- | --- | --- |
| 2 | NM_009134.3 | 6413 | NP_033160.2 | 1957 |
| 1 | NM_001205321.1 | 6416 | NP_001192250.1 | 1958 |
| X4 | XM_017313223.1 | 6696 | XP_017168712.1 | 1958 |
| X1 | XM_017313221.1 | 5531 | XP_017168710.1 | 1437 |
| X3 | XM_036154738.1 | 4980 | XP_036010631.1 | 1194 |
| X2 | XM_017313222.3 | 4144 | XP_017168711.1 | 1195 |
