# Supplementary material for: Transiently Nav1.8-expressing neurons are capable of sensing noxious stimuli in the brain
Source: Front Cell Neurosci. 2022 Aug 29;16:933874. doi: 10.3389/fncel.2022.933874 (PMC9464809; doi:10.3389/fncel.2022.933874)
Supplement: Supplementary Table S3 — Brain regions and respective anterior-posterior coordinates (in mm) where Nav1.8 neurons were quantified. [file Table_3.DOCX]

**Supplementary Table 3.** Brain regions and respective anterior-posterior coordinates (in mm) where Nav1.8 neurons were quantified

|  | +1.42 | +1.04 | +0.62 | +0.14 | -0.08 | -0.48 | -1.05 | -1.55 | -2.15 | -2.55 |
| --- | --- | --- | --- | --- | --- | --- | --- | --- | --- | --- |
| LSr |  |  |  |  |  |  |  |  |  |  |
| LSc |  |  |  |  |  |  |  |  |  |  |
| LSv |  |  |  |  |  |  |  |  |  |  |
| mHY |  |  |  |  |  |  |  |  |  |  |
| LHA |  |  |  |  |  |  |  |  |  |  |
| CEA |  |  |  |  |  |  |  |  |  |  |
| CEAl |  |  |  |  |  |  |  |  |  |  |
| MEAad |  |  |  |  |  |  |  |  |  |  |
| MEAav |  |  |  |  |  |  |  |  |  |  |
| BMAp |  |  |  |  |  |  |  |  |  |  |
| COApl |  |  |  |  |  |  |  |  |  |  |
| PAA |  |  |  |  |  |  |  |  |  |  |
| STRd |  |  |  |  |  |  |  |  |  |  |
| BST |  |  |  |  |  |  |  |  |  |  |
| PAG |  |  |  |  |  |  |  |  |  |  |
